# Subclavian-Carotid Bypass as a Solution to Recurrent Carotid Artery Stenosis Post Endarterectomy and Transfemoral Stenting

**DOI:** 10.7759/cureus.63087

**Published:** 2024-06-25

**Authors:** Pranav Balakrishnan, Jamie Anderson, Christina M Arcand, Matthew Krantz, James C Kitchen

**Affiliations:** 1 General Surgery, Marshall University Joan C. Edwards School of Medicine, Huntington, USA; 2 Vascular Surgery, Marshall University Joan C. Edwards School of Medicine, Huntington, USA

**Keywords:** post-carotid endarterectomy, subclavian-carotid bypass, carotid bypass, carotid stent, carotid in-stent restenosis, endovascular therapy, recurrent carotid stenosis, carotid artery stenosis, general and vascular surgery

## Abstract

We present the case of an 80-year-old man who underwent a subclavian-to-distal internal carotid artery bypass with a reversed saphenous vein due to symptomatic in-stent restenosis, following a carotid endarterectomy 20 years ago and carotid artery stenting 10 years ago.

The patient presented with right-sided hemiparesis and dysarthria. Imaging suggested in-stent restenosis of the internal carotid artery stent. He was also found to have stenosis of the common carotid artery origin stent. An initial transfemoral attempt by interventional radiology was unsuccessful. Due to the stenosed common carotid artery origin stent, a common carotid-to-internal carotid artery bypass was not feasible. Therefore, a subclavian-distal carotid artery bypass with a reversed saphenous vein was performed.

He did well in the postoperative period and has been seen in the clinic since. Surveillance ultrasound demonstrated a patent graft with non-stenotic proximal and distal anastomoses. We include an in-depth review of the management of recurrent carotid artery stenosis as well.

## Introduction

Carotid artery stenosis is a common cause of ischemic stroke that is effectively treated by carotid endarterectomy (CEA) or carotid artery stenting (CAS) [1,2]. Restenosis is a known long-term complication following both CEA and CAS [3]. Several options are available to manage carotid artery restenosis including repeat CAS, CEA, and carotid bypass [4]. Our case describes a unique situation where the patient underwent a CEA, had restenosis, and underwent transfemoral stenting, only to have in-stent restenosis (ISR) again, and presented to us with a transient ischemic attack. At this point, the only option available to us was a subclavian carotid bypass.

## Case presentation

This is the case of an 80-year-old gentleman with a history of aortic stenosis having undergone transcatheter aortic valve replacement and hypertension, who presented with right-sided hemiparesis and dysarthria. Vascular surgery was consulted four days after presentation, at which point, the patient’s weakness had improved, as had his dysarthria. Of note, the patient has a history of bilateral carotid endarterectomies done 20 years prior and a transfemoral left common carotid artery (CCA) and left internal carotid artery (ICA) stents done 10 years prior. He continues to smoke but has decreased the quantity to one-half pack per day.

**Figure 1:**
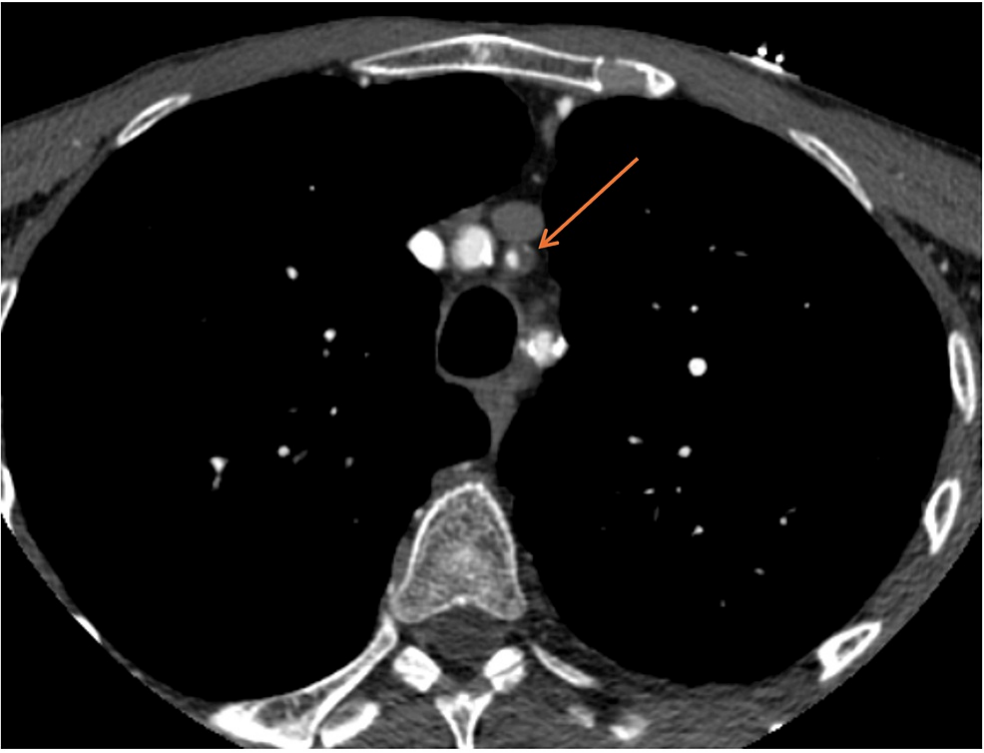
Axial view of a CT angiogram depicting in-stent restenosis of left common carotid artery stent (arrow).

**Figure 2:**
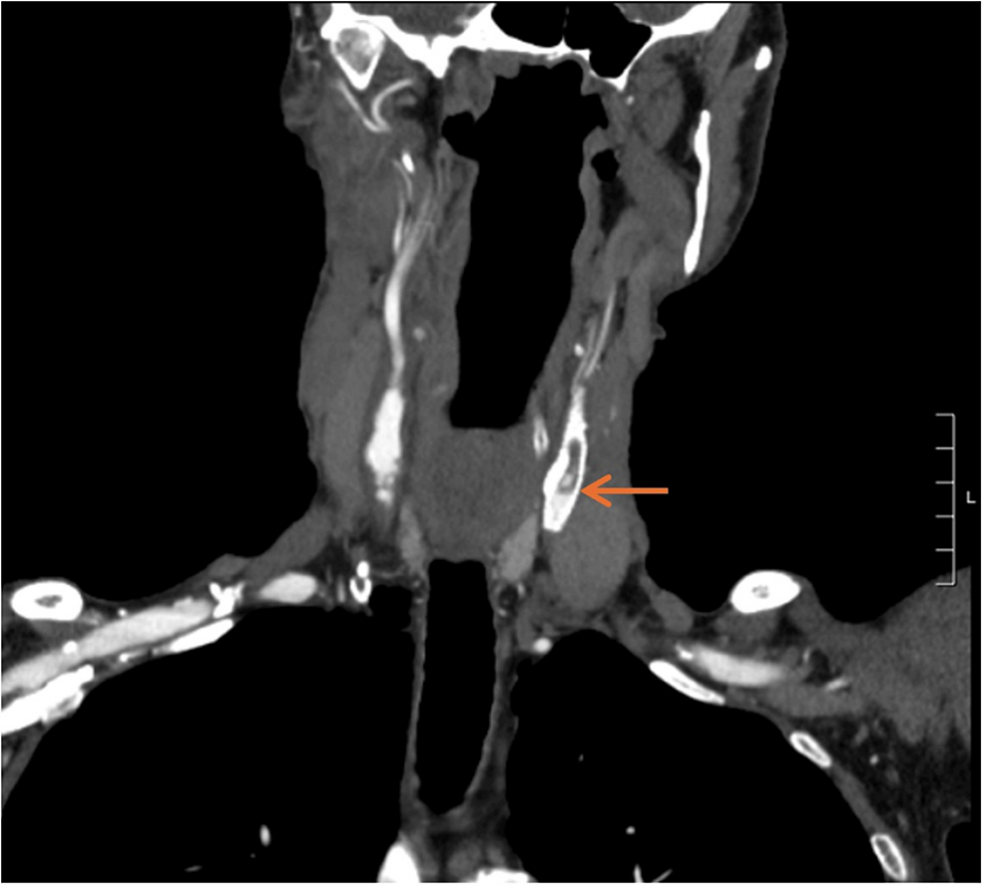
Coronal view of a CT angiogram depicting in-stent restenosis of left common carotid artery stent (arrow).

**Figure 3:**
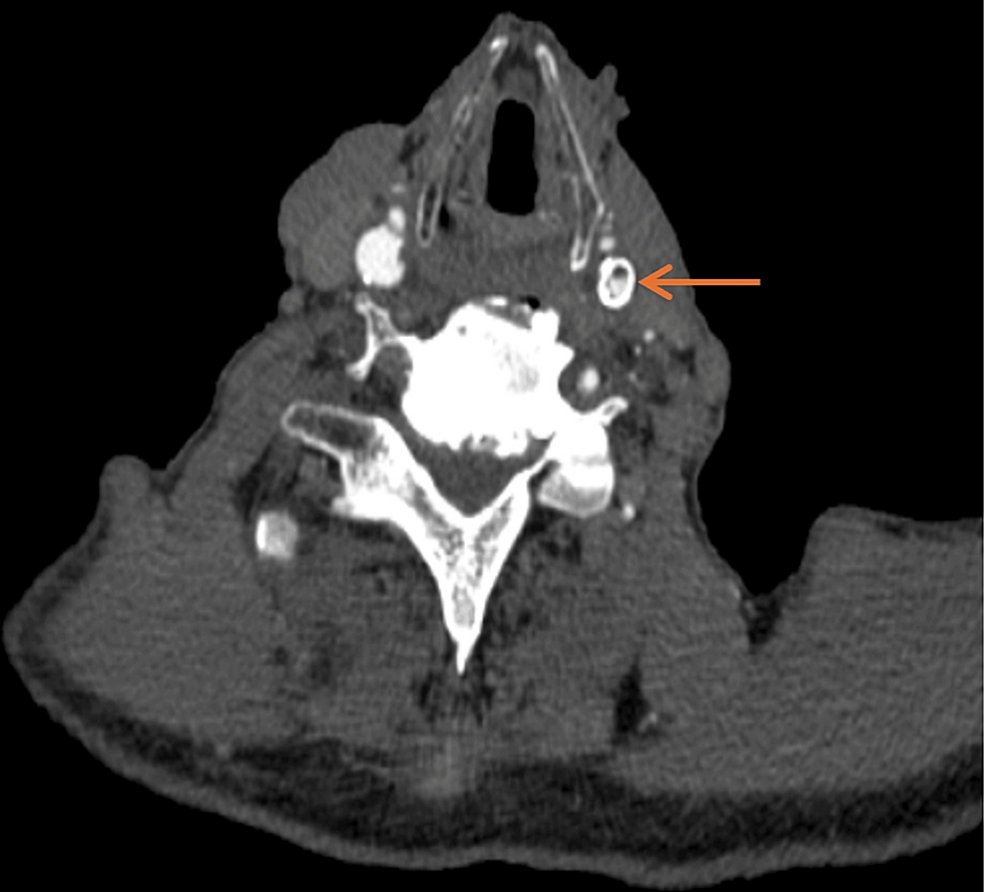
Axial view of a CT angiogram depicting in-stent restenosis of left internal carotid artery stent (arrow).

CT-angiogram on presentation revealed greater than 90% ISR of a left CCA orifice stent and 60% stenosis of a left ICA stent, calculated in accordance with North American Symptomatic Carotid Endarterectomy Trial (NASCET) criteria (Figures 1-3). MRI revealed acute left-sided infarcts, confirming the diagnosis of symptomatic ISR.

**Figure 4:**
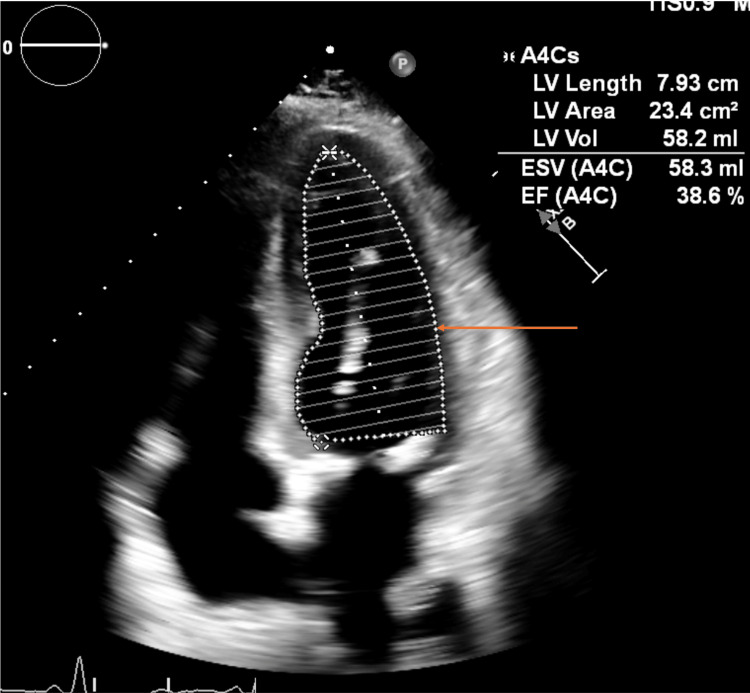
Apical four-chamber view of the heart on echocardiogram, with left ventricular (arrow) ejection fraction being calculated.

**Figure 5:**
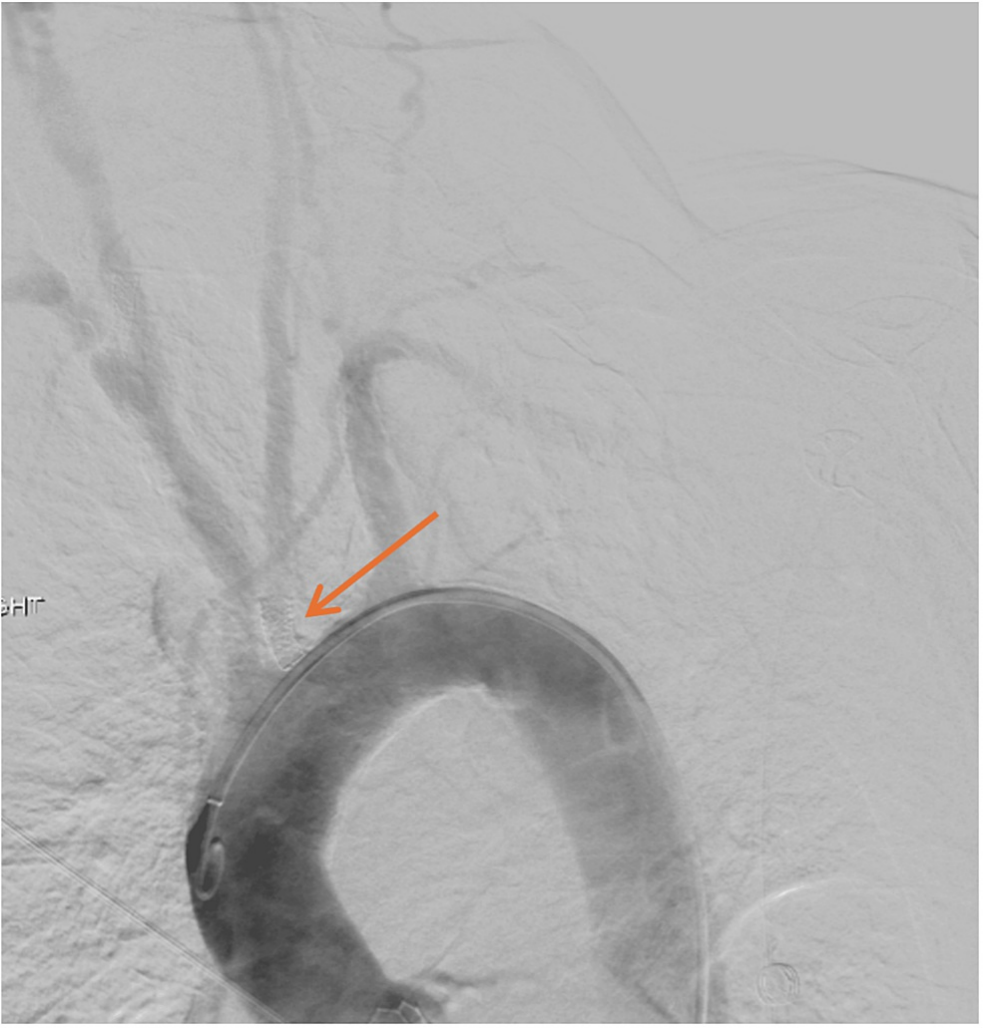
Thoracic aortogram with left common carotid artery stent and in-stent restenosis (arrow).

Given that the patient has an extensive cardiac history as well, including a recent transcatheter aortic valve replacement one year prior, and an ejection fraction of 38.6% (Figure 4), we consulted interventional radiology to attempt a transfemoral intervention. Unfortunately, this intervention was unsuccessful. While a guidewire was advanced past the lesions in the left CCA and ICA, a balloon or catheter would not cross the stenotic segments. A selective common carotid arteriogram during the procedure showed near-total occlusion of the left CCA stent (Figure 5). 

Given the symptomatic ISR, we planned to proceed with a left subclavian-internal carotid bypass with the exclusion of the stents to decrease his risk of stroke. Of note, the patient remained on dual anti-platelet therapy with aspirin and clopidogrel for the entire perioperative period.

**Figure 6:**
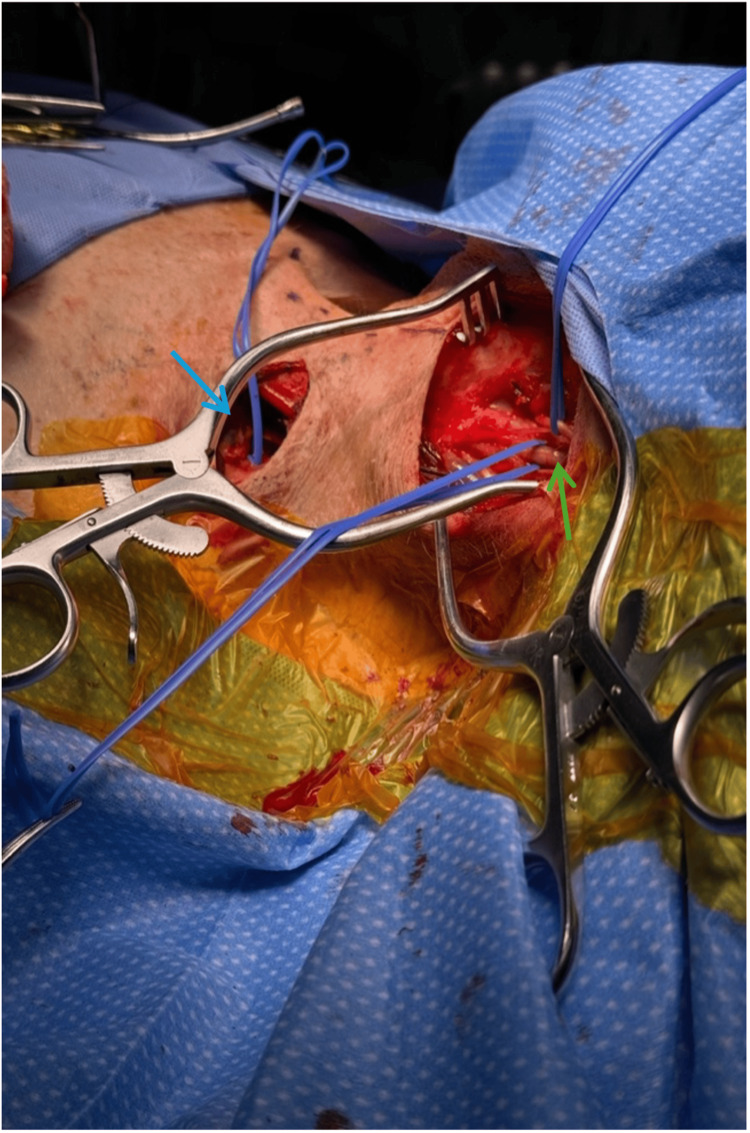
Intra-operative exposure with left supraclavicular incision (blue arrow) and left neck incision with internal carotid artery (green arrow).

Intra-operatively, the left great saphenous vein (GSV) appeared to be of good caliber on ultrasound and a good size match to the ICA. A supraclavicular incision was made, and dissection was carried out down to the subclavian artery by transecting the anterior scale muscle along the first rib, with care taken to preserve the phrenic nerve. Next, attention was turned to exposing the left ICA through an incision just anterior to the sternocleidomastoid curving cephalad toward the mastoid process. The left neck had dense scar tissue given the prior procedures. With great care, dissection was carried out down to the ICA, preserving the vagus and hypoglossal nerves. Our patient appeared to have had a primary repair following his CEA. A tunnel was then created between the supraclavicular and neck incision (Figure 6). Heparin was administered to therapeutic levels. 

**Figure 7:**
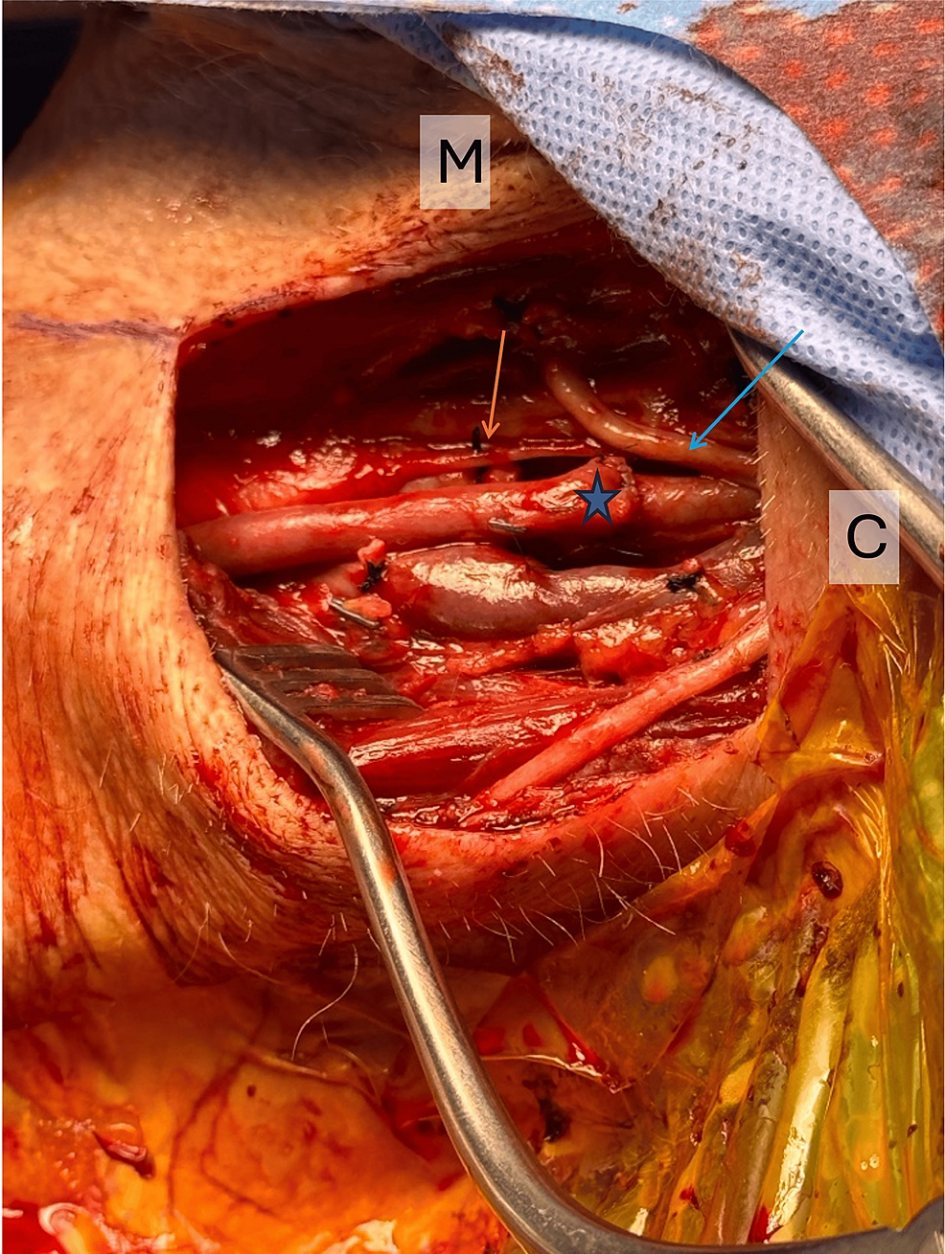
Left neck incision with ligated proximal internal carotid artery (orange arrow), reversed saphenous vein (star), and hypoglossal nerve (blue arrow). C: Cephalad, M: Medial

Proximal and distal clamps were applied to the subclavian artery, and the proximal anastomosis was completed between the subclavian artery and the reversed GSV in an end-to-side fashion. The GSV was then tunneled anterior to the phrenic nerve, under the sternocleidomastoid, into the neck incision. The proximal ICA was ligated and transected just distal to the Left ICA stent to exclude it from circulation. The distal anastomosis was then performed between the distal ICA and reversed GSV in an end-to-end fashion (Figure 7). A 15 French Blake drain was left, and all the incisions were closed in a layered fashion. The patient had no neurological deficits once awakened from general anesthesia.

The patient did well postoperatively. Once on a regular diet, there was no evidence of a chyle leak in the Blake drain. On postoperative day 3, the drain was removed. The patient worked with physical therapy and was found to have no acute rehabilitation needs. He was discharged on postoperative day 4. He was seen in the clinic two weeks postoperatively. There was no evidence of a surgical site infection or neurological deficit. Surveillance ultrasound demonstrated a patent graft with non-stenotic proximal and distal anastomoses.

## Discussion

The NASCET established the role of CEA in treating symptomatic high-grade carotid stenosis [1]. Similarly, the carotid revascularizations endarterectomy versus stenting trial (CREST) was carried out to evaluate CAS as a modality to treat carotid stenosis in comparison to CEA. During the perioperative period, it was found that CAS was associated with a higher incidence of stroke, while CEA was associated with an increased risk of myocardial infarction. In the four-year period of follow-up, however, the risk of post-procedural stroke, myocardial infarction, or death was not statistically different [2].

Bonati et al. secondarily analyzed restenosis and the risk of stroke after CAS or CEA for symptomatic carotid stenosis in the International Carotid Stenting Study (ICSS). They found that >50% restenosis was seen in 40.7% of patients in the CAS group versus 29.6% in the CEA group at five years. Severe stenosis (>70%) was seen in 10.6% of the CAS group and 8.5% of the CEA group at five years. Not surprisingly, patients with at least moderate restenosis had a higher risk of ipsilateral stroke compared to those without restenosis in the overall patient population and those in the CEA group [3]. In the case of our patient in question, he had a CEA 20 years prior with subsequent restenosis, followed by CAS 10 years prior, which also had ISR.

Seven hundred ninety patients who underwent endarterectomy with patch angioplasty, primary closure, and eversion endarterectomy were studied for rates of long-term restenosis over a period of five years. Primary closure had a higher risk of >50% restenosis than patch closure (HR 1.45, 95% CI 1.06-1.98, p = 0.019). There was no statistically significant difference between the two with regards to high-grade restenosis (>70%), or in the case of eversion endarterectomy. Ipsilateral stroke in the post-procedure period was not common [5]. Intraoperatively, our patient appeared to have had a primary repair following his CEA.

A meta-analysis by Texakalidis et al. highlighted diabetes, dyslipidemia, female gender, renal failure, hypertension, and smoking were associated with an increased risk of restenosis. Patch endarterectomy and symptomatic status and index procedure decreased the risk of carotid restenosis [6].

A systematic review by Huang et al. evaluated interventions performed for carotid ISR in 1,359 patients. 66.3% of the patients were treated with repeat CAS, 17.5% underwent transluminal angioplasty, 14.3% underwent CEA, and 1.5% had a carotid artery bypass performed. The postoperative rates of stroke and TIA were similar [4].

Owens et al. described surgical interventions performed for two patients in their experience who developed restenosis following CAS. One patient received an ICA-to-external carotid artery transposition, and the other patient received a common carotid artery-to-ICA bypass with a reversed saphenous vein [7]. In our case, CAS was attempted, which failed. With regards to bypass, the presence of the stenosed CCA origin stent precluded performing a CCA to ICA bypass. A subclavian-to-ICA bypass was the next logical option.

There is a lack of research on autologous veins versus artificial grafts such as polytetrafluoroethylene (PTFE) as a conduit for subclavian-carotid bypasses. Data are available in favor of PTFE use for carotid-subclavian bypasses in the setting of arm ischemia and vertebrobasilar insufficiency [8,9]. In our patient, given the size match of the GSV and the distal ICA, we elected to perform the bypass with a reversed GSV.

## Conclusions

Our patient presented with symptomatic ISR following a CEA 20 years ago and CAS 10 years ago. Transfemoral intervention was attempted by interventional radiology at this point and was unsuccessful. With regards to options for a bypass, the presence of the stenosed CCA origin stent precluded performing a CCA to ICA bypass. As seen in our case, if encountered with this unique situation, a subclavian-to-distal ICA bypass is a reasonable option.

Symptomatic ISR is a complex disease process, where a multi-disciplinary approach is essential to arrive at the appropriate treatment for the patient and the best possible outcome. Our case describes the various possible alternatives and the extensive considerations that go into treating recurrent carotid artery stenosis.

## References

[1] Barnett HJ, Taylor DW, Haynes RB (1991). Beneficial effect of carotid endarterectomy in symptomatic patients with high-grade carotid stenosis. N Engl J Med.

[2] Brott TG, Hobson RW 2nd, Howard G (2010). Stenting versus endarterectomy for treatment of carotid-artery stenosis. N Engl J Med.

[3] Bonati LH, Gregson J, Dobson J (2018). Restenosis and risk of stroke after stenting or endarterectomy for symptomatic carotid stenosis in the International Carotid Stenting Study (ICSS): secondary analysis of a randomised trial. Lancet Neurol.

[4] Huang H, Wu L, Guo Y, Zhang Y, Zhao J, Yu Z, Luo X (2021). Treatment of the carotid in-stent restenosis: a systematic review. Front Neurol.

[5] Cheng SF, Richards T, Gregson J, Brown MM, de Borst GJ, Bonati LH (2021). Long term restenosis rate after carotid endarterectomy: comparison of three surgical techniques and intra-operative shunt use. Eur J Vasc Endovasc Surg.

[6] Texakalidis P, Tzoumas A, Giannopoulos S (2019). Risk factors for restenosis after carotid revascularization: a meta-analysis of hazard ratios. World Neurosurg.

[7] Owens EL, Kumins NH, Bergan JJ, Sparks SR (2002). Surgical management of acute complications and critical restenosis following carotid artery stenting. Ann Vasc Surg.

[8] AbuRahma AF, Robinson PA, Jennings TG (2000). Carotid-subclavian bypass grafting with polytetrafluoroethylene grafts for symptomatic subclavian artery stenosis or occlusion: a 20-year experience. J Vasc Surg.

[9] Ziomek S, Quinones-Baldrich WJ, Busuttil RW (1986). The superiority of synthetic arterial grafts over autologous veins in carotid-subclavian bypass. J Vasc Surg.

